# Errata

**Published:** 2014-11-21

## Vol. 63, No. 46

In the report, “Ebola Epidemic — Liberia, March–October 2014,” which was first published as an *MMWR* Early Release on November 14, 2004, multiple errors occurred.

The list of authors and their affiliations should read as follows: Tolbert Nyenswah^1^, Miatta Fahnbulleh^1^, Francis Ketah^1^, Moses Massaquoi^1^, Thomas Nagbe^1^, Luke Bawo^1^, James Dorbor Falla^1^, Henry Kohar^1^, Alex Gasasira^2^, Pierre Nabeth^2^, Heather Popowitz^3^, Sheldon Yett^3^, Lindis Hurum^4^, Laurence Sailly^4^, Sean Casey^5^, Benjamin Espinosa^6^, Andrea McCoy^6^, Heinz Feldman^7^, Lisa Hensley^7^, Mark Baily^8^, Justin Pendarvis^9^, Barry Fields^10^, Terrence Lo^10^, Jin Quin^10^, John Aberle-Grasse^10^, Kim Lindblade^10^, Josh Mott^10^, Lucy Boulanger^10^, Athalia Christie^10^, Susan Wang^10^, Joel Montgomery^10^, Frank Mahoney^10^ (Author affiliations at end of text).

^1^Ministry of Health and Social Welfare, Liberia; ^2^World Health Organization; ^3^United Nations Children’s Fund; ^4^Médecins Sans Frontières; ^5^International Medical Corps; ^6^U.S. Navy; ^7^National Institutes of Health; ^8^U.S. Army Medical Research Institute of Infectious Diseases; ^9^U.S. Agency for International Development; ^10^CDC

In the fifth paragraph, the third sentence should read as follows: “**After aggressive response** in Lofa, the county experienced multiple outbreak waves, with a peak in case counts between late July and late September (Figure 1).”

In the sixth paragraph, the fourth sentence should read as follows: “As of November 8, MoHSW reported 329 health care workers infected with Ebola**, including 157 who died**.”

In the seventh paragraph, the third sentence should read as follows: “The Firestone company established a **10-bed unit** in April in Margibi county (*4*).”

In the Discussion, the fifth sentence should read, “However, progress of control efforts is tenuous **as situation reports in the past 2 weeks suggest a leveling off of case counts and outbreaks in new areas.”**

## Vol. 63, No. 45

In this issue, the date in the title of an announcement was incorrect. The title should read, “World Day of Remembrance for Road Traffic Victims — November 16, **2014**.”

## Vol. 63, No. 44

In the report, “Declines in Pneumonia Hospitalizations of Children Aged <2 Years Associated with the Use of Pneumococcal Conjugate Vaccines — Tennessee, 1998–2012,” in Table 2, under the heading and subheading PCV13 years compared with pre-PCV7 years^†^, % change in rates, the value should read, -**72**.

